# *In Vitro* Propagation and Reintroduction of the Endangered *Renanthera imschootiana* Rolfe

**DOI:** 10.1371/journal.pone.0110033

**Published:** 2014-10-28

**Authors:** Kunlin Wu, Songjun Zeng, Danni Lin, Jaime A. Teixeira da Silva, Zhaoyang Bu, Jianxia Zhang, Jun Duan

**Affiliations:** 1 Key Laboratory of Plant Resources Conservation and Sustainable Utilization, South China Botanical Garden, Chinese Academy of Sciences, Guangzhou, China; 2 Key Laboratory of South China Agricultural Plant Molecular Analysis and Gene Improvement, South China Botanical Garden, Chinese Academy of Sciences, Guangzhou, China; 3 Kagawa-ken, Japan; 4 Flower Research Institute of Guangxi Academy of Agricultural Sciences, Nanning, China; Huazhong University of Science & Technology(HUST), China

## Abstract

*Renanthera imschootiana* Rolfe is an endangered tropical epiphytic orchid that is threatened with extinction due to over-collection and the loss of suitable habitats. *In vitro* propagation is a useful way to mass produce plants for re-establishment in the wild and for commercial propagation. Seeds collected 150 days after pollination (DAP) were the optimum stage for *in vitro* culture. Seed germination reached 93.1% on quarter-strength MS (i.e., MS containing a quarter of macro- and micronutrients) medium containing 0.5 mg l^−1^ α-naphthaleneacetic acid (NAA), 20% coconut water (CW), 1.0 g l^−1^ peptone, 10 g l^−1^ sucrose and 1.0 g l^−1^ activated charcoal (AC). Quarter-strength MS medium supplemented with 1.0 mg l^−1^ BA, 0.5 mg l^−1^ NAA, 1.0 g l^−1^ peptone, 10 g l^−1^ sucrose and 20% CW was suitable for the sub-culture of protocorm-like bodies (PLBs) in which the PLB proliferation ratio was 2.88. Quarter-strength MS medium containing 1.0 mg l^−1^ NAA, 1.0 g l^−1^ peptone, 100 g l^−1^ banana homogenate (BH), and 1.0 g l^−1^ AC was suitable for plantlet formation and 95.67% of plantlets developed from PLBs within 60 days of culture. Hyponex N016 medium **s**upplemented with 0.5 mg l^−1^ NAA, 1.0 g l^−1^ peptone, 20 g l^−1^ sucrose, 150 g l^−1^ BH, and 1.0 g l^−1^ AC was suitable for the *in vitro* growth of plantlets about 2-cm in height. Plantlets 3-cm in height or taller were transplanted to Chilean sphagnum moss, and 95% of plantlets survived after 60 days in a greenhouse. Three hundred transplanted of seedlings 360-days old were reintroduced into three natural habitats. Highest percentage survival (79.67%) was observed in Yuanjiang Nature Reserve two years after reintroduction, followed by Huolu Mountain forest park (71.33%). This protocol is an efficient means for the large-scale propagation and *in vitro* and *in vivo* germplasm conservation of *R. imschootiana*.

## Introduction

*Renanthera* is a genus of large scrambling monopodial epiphytic, lithophytic and terrestrial species found in India, China, Vietnam, New Guinea, Malaysia, Indonesia, the Philippines and the Solomon Islands [1]–[2]. Approximately 20 species of this genus produce a branched inflorescence containing numerous flowers ranging in color from yellow and orange to red and their flowers possess large lateral sepals [2]–[3]. *R. imschootiana* is the only species in the genus listed in Appendix I of the regulations formulated by the Committee for International Trade in Endangered Species Wild Fauna and Flora [4]. This listing is a result of over-collection for use as ornamental plants or as breeding parents and loss of suitable habitats caused exclusively by human activity in response to the trade of this orchid [5]. *R. imschootiana* is an extremely rare and endangered tropical epiphytic orchid that is only distributed in Yuanjian County, Yunnan, China and in Myanmar, India and Vietnam [1]–[2], [6]. This species has considerable horticultural value, particularly for its bright colors and long-lasting flowers, and is a progenitor of many outstanding hybrids, including *R.* Tom Thumb, *Rendopsis* Hiiaka and *Renanthopsis* Jan Stokes [6]–[7]. The current (22 September, 2014) value of an adult *R. imschootiana* plant is 100 RMB (1 USD = 6.134 CNY; www.xe.com). Currently, 78 hybrids use *R. imschootiana* as parents, 46 as seed parents and 32 as pollen parents, assessed until September 22, 2014 [8].

Asymbiotic seed germination and micropropagation of orchid seeds are efficient methods to propagate orchids on a large scale [9]–[10]. In *Renanthera*, only five protocols for *in vitro* propagation have been described in the literature. Goh and Tan [11] reported plant regeneration from young leaves of mature plants of *R.* Ammani (*Vanda* Josephine Van Brero×*R. storiei*). Seeni and Latha [6] reported an *in vitro* method that could regenerate large numbers of phenotypically uniform plants from the basal parts of the leaves of flowering *R. imschootiana* plants. Lin et al. [12] reported asymbiotic germination *in vitro* of *R. imschootiana* in a preliminary study. Rajkumar and Sharma [13] propagated the intergeneric hybrid of *R. imschootiana* and *Vanda coerulea* by *in vitro* seed germination. Wu et al. [5] could regenerate *R.* Tom Thumb ‘Qilin’ (*R. imschootiana*×*R. monachica*) from leaf explants.

The goal of this research was to develop effective protocols for the propagation of *R. imschootiana* from seed to meet commercial needs and to re-establish plants back into the wild. This objective was achieved by asymbiotic seed germination, *in vitro* seedling culture, greenhouse acclimatization, and the re-establishment of *in vitro*-derived plants in the wild at three locations.

## Materials and Methods

### Seed source and sterilization

Two-hundred *R. imschootiana* plants collected from Yuanjian County, Yunnan, China were potted in substrate mixture 2 [containing commercial sand for orchids, sieved peat and shattered fir bark (2∶1∶1; v/v)] in a greenhouse in South China Botanical Garden, Guangzhou, China. Based on initial trials, fruit setting percentage and seed viability from self-pollination were not significantly different to cross-pollination: fruit setting percentage exceeded 90% in both cases. Therefore, self-pollination was employed in which the flowers from adult plants were labeled and artificially self-pollinated by transferring pollen onto the stigma of the same flower as they became fully opened. Ten capsules from independent plants were collected at different developmental stages every 30 days from 30 to 240 days after pollination (DAP). Capsules older than 270 DAP usually split. Capsules were surface sterilized by dipping into 75% (v/v) ethanol for 2 min, followed by agitation for 15 min in a sodium hypochlorite (NaOCl) solution containing 2% available chlorine and 0.05% (v/v) Tween-20, after which the capsules were rinsed five times with sterile distilled water. All seeds were removed from 10 capsules to calculate the mean number of seeds per capsule and the dry weight of individual seeds as follows: 1) all dry seeds in a capsule were weighed on a two-digit electronic balance; 2) one-tenth (w/w) of dry seeds were placed into 10 ml of water and the number of seed in 1 ml of water was calculated under a Leica S8APO (Wetzlar, Germany); 3) the number of seeds in each capsule was calculated by the number of seeds in 1 ml of water ×1000 while individual mean seed dry weight was calculated by seed dry weight in a capsule/number of seeds. Ten seeds were also observed by scanning electron microscopy (SEM). SEM samples (20 seeds) were fixed in 2.5% glutaraldehyde for 12 h and dehydrated in an ethanol series (30%→50%→70%→80%→90%→100%) for 10 min in each step, then freeze dried in a JFD-310 vacuum freeze dryer for 2.5 h. Seeds were sputter coated with gold and observed with a JSM-6360LV scanning electron microscope and digital pictures were recorded [14].

### Effect of basal media on germination *in vitro*

To determine the influence of basal media on seed germination and subsequent protocorm development, 10 capsules disinfected at 150 DAP were cut open vertically with a sterile scalpel, and the seeds were placed onto nine basal sowing media in which ratios represent the fraction of macro- and micro-nutrients: 1) one-eighth-strength Murashige and Skoog (1/8 MS) [15]; 2) quarter-strength MS (1/4 MS); 3) half-strength MS (1/2 MS); 4) MS; 5) Knudson's C (KC) [16]; 6) Vacin and Went (VW) [17]; 7) Robert-Ernst (RE) [18]; 8) Thomale GD [19]; 9) Hyponex N016 [14]; 10) Hyponex N026 [20]. All 10 media were supplemented with 0.5 mg l^−1^ α-napthaleneacetic acid (NAA; Sigma Chemical Co., St. Louis, USA), 10 g l^−1^ sucrose and 1.0 g l^−1^ activated charcoal (AC) in which all concentrations were optimized in initial trials.

**Figure 1:**
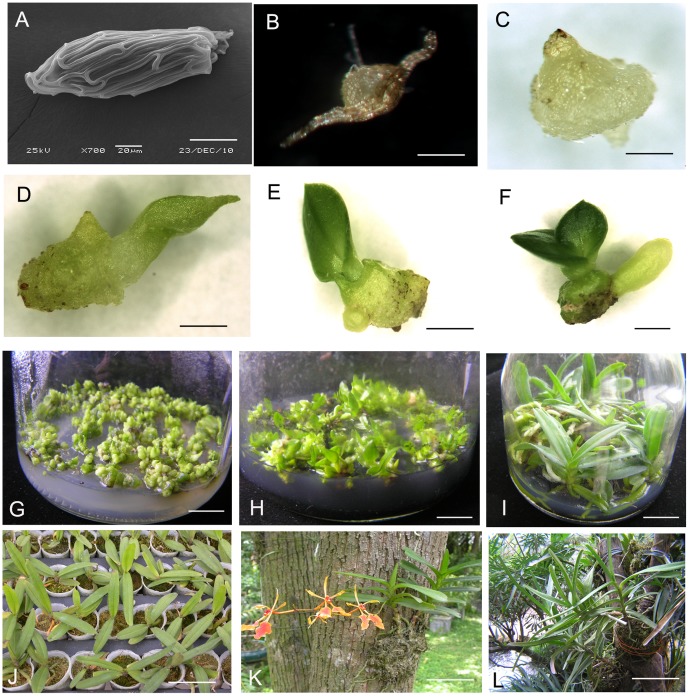
*In vitro* seed culture, seedling development and reintroduction of *Renanthera imschootiana* Rolfe. (A) Stage 0, seed under scanning electron microscopy, ungerminated. (B) Stage 1, testa ruptured. (C) Stage 2, appearance of rhizoids. (D) Stage 3, emergence and elongation of first leaf. (E) Stage 4, one leaf and root present. (F) Stage 5, presence of two or more leaves. (G) Proliferation of PLBs on quarter-strength MS (1/4 MS) medium supplemented with 1.0 mg l^−1^ BA, 1.0 mg l^−1^ NAA, 1.0 g l^−1^ peptone and 20% CW. (H) Differentiation of PLBs on 1/4 MS medium supplemented with 1.0 mg l^−1^ NAA, 1.0 g l^−1^ peptone, 100 g l^−1^ BH, and 1.0 g l^−1^ AC. (I) Development of seedlings on Hyponex N016 medium supplemented with 0.5 mg l^−1^ NAA, 1.0 g l^−1^ peptone, 150 g l^−1^ BH, and 1.0 g l^−1^ AC. (J) Transplanted plantlets 6 months after acclimatization in the greenhouse. (K) Reintroduced flowering plantlets from *in vitro*-derived seedlings on the trunk of *Pinus massoniana* on Huolu Mountain, Guangzhou. (L) Reintroduced plantlets from *in vitro*-derived seedlings at the Orchids Garden, South China Botanical Garden. Bars: (A) 50 µm, (B) 100 µm, (C) 0.05 mm, (D) 0.35 mm, (E) 0.40 mm, (F) 0.60 mm, (G, H) 3.0 cm, (I) 4.0 cm, (J, K) 5.0 cm, (L) 3.0 cm.

For each treatment, *ca.* 300 seeds were cultured in a 500-ml culture flask containing 90 ml of medium. All experiments consisted of three independent replicates with 10 culture flasks per replicate from 10 capsules. Cultures were observed every 30 days for signs of germination and subsequent protocorm development under a Leica S8APO (Wetzlar, Germany). Developmental stages (Table 1, Fig. 1A–F) were adapted from Zeng et al. [14] The percentage of germinating seed/protocorms at each developmental stage was calculated by dividing the number of seed/protocorms in each stage by the total number of cultured seeds in each flask ×100. Germination was considered to have occurred only if a swollen embryo was present and if the testa had ruptured (Stage 1). The start of germination was calculated when round or ovoid hyaline embryos (viable embryos) were present [14].

**Table 1:** Seed germination and seedling developmental growth stages of *Renanthera imschootiana* (modified from Zeng et al., 2012).

Stage	Description
0	Ungerminated seed with embryo and unruptured testa
1	Ruptured testa by enlarging embryo ( = germination)
2	Appearance of shoot ( = protomeristem) and/or rhizoids
3	Emergence and elongation of first leaf
4	One leaf and one or more roots present
5	Presence of two or more leaves, roots present ( = seedling)

### Effect of seed maturity degree on germination *in vitro*

To determine the influence of the degree of seed maturity on seed germination, *R. imschootiana* seed capsules were collected continuously every 30 days from 30 to 240 DAP. According to initial trials, 1/4 MS medium supplemented with 0.5 mg l^−1^ NAA, 10 g l^−1^ sucrose, 20% coconut water (CW, v/v), 1.0 g l^−1^ peptone, and 1.0 g l^−1^ AC was the most suitable for seed germination. Therefore, at each collection date, seeds were placed on this medium. The percentage of seed at each developmental stage was calculated by dividing the number of seed/protocorms at 75 days by the total number of seeds in each flask ×100. The total number of seeds in each flask was calculated under a Zeiss stereomicroscope.

### Effect of sucrose concentration on seed germination

To determine the influence of sucrose concentration on seed germination, 1/4 MS containing 0, 5, 10, 15, 20, 25, 30 or 35 g l^−1^ sucrose, 0.5 mg l^−1^ NAA, 20% CW, 1.0 g l^−1^ peptone, and 1.0 g l^−1^ AC was tested. The percentage of germinating seed was calculated by dividing the number of seed/protocorms at 75 days by the total number of seeds in each flask ×100 under a Leica S8APO microscope.

### Effect of organic amendments on seed germination

To determine the influence of organic amendments on seed germination and subsequent protocorm development, 1/4 MS containing 10%, 15%, 20%, or 25% CW, or 50, 100, or 150 g l^−1^ ripe banana homogenate (BH) and potato homogenate (PH), or 0.5, 1.0, or 1.5 g l^−1^ tryptone and peptone, or 20% CW combined with 0.5, 1.0, or 1.5 g l^−1^ peptone. All experiments consisted of three independent replicates with 10 culture flasks per replicate. The percentage of germinating seed was calculated by dividing the number of seed/protocorms at 75 days by the total number of seeds in each flask ×100 under a Leica S8APO microscope.

### Effect of BA concentration on PLB proliferation

The effect of 6-benzyladenine (BA; 0, 0.2, 0.5, 1.0, 1.5, 2.0, or 2.5 mg l^−1^) on protocorms or protocorm-like body (PLB) proliferation was measured on 1/4 MS medium supplemented with 0.5 mg l^−1^ NAA, 1.0 g l^−1^ peptone, 10 g l^−1^ sucrose, and 20% CW at 60-day intervals for each subculture under a Leica S8APO microscope. Twelve subcultures were performed over a total of approximately 2 years. PLB proliferation efficiency was calculated as the ratio of the number of PLBs newly formed divided by the number of PLBs incubated only in the 8^th^ sub-culture. Thirty PLBs (1.5 mm in diameter) were cultured in each flask, and each experiment consisted of three independent replicates with 10 culture flasks per replicate.

### Effect of NAA and banana homogenate concentration on PLB differentiation

PLB differentiation was assessed on 1/4 MS medium containing 1.0 g l^−1^ peptone, 10 g l^−1^ sucrose, 10% CW and 1.0 g l^−1^ AC and NAA at 0, 0.5, 1.0, 1.5 or 2.0 mg l^−1^, or BH at 50, 100, 150, or 200 g l^−1^, or 0.5, or 1.0 mg l^−1^ NAA in combination with 50, 100, or 150 g l^−1^ BH under a Leica S8APO microscope. The efficiency of PLB differentiation was calculated as the ratio of the number of shoots formed divided by the number of incubated PLBs only in the 8^th^ sub-culture. Thirty PLBs were cultured per 500-ml flask, and each experiment consisted of three independent replicates with 10 culture flasks per replicate.

### Effect of NAA and banana homogenate concentration on growth of plantlets *in vitro*

Following initial trials, Hyponex N016 medium was found to be most suitable for the *in vitro* growth of plantlets among all tested basal media. Plantlets about 2 cm in height with two roots and three leaves were used to test the effect of NAA and BH concentration on plantlet growth. The status of plantlet growth (shoot height, number of roots, length of the longest root, fresh weight/plantlet and vitality, labeled by +, ++, +++ represent poor, normal, and good growth, respectively) was assessed on plantlets growing on Hyponex N016 medium containing 1.0 g l^−1^ peptone, 20 g l^−1^ sucrose, 10% CW and 1.0 g l^−1^ AC and NAA at 0, 0.5, 1.0, 1.5, or 2.0 mg l^−1^, BH at 50, 100, 150, or 200 g l^−1^, or 0.5, or 1.0 mg l^−1^ NAA in combination with 100, 150, or 200 g l^−1^ BH. All experiments consisted of three independent replicates with 10 culture flasks per replicate, with 20 plantlets in each flask.

### Greenhouse acclimatization

*In vitro* propagated plantlets 3-cm in height or taller were transferred to natural greenhouse conditions to acclimatize for 10 days, then transplanted to five different supporting substrates between April and June: 1) each plantlet was fixed on an 8 cm×15 cm fir bark block held within nylon wire; 2) each plantlet was potted in Chilean sphagnum moss held in a 5-cm diameter polyethylene planting bag with holes for water drainage; 3) each plantlet was fixed on a fir bark block in which roots were packaged in Chilean sphagnum moss; 4) each plantlet was potted in substrate mixture 1 containing commercial sand for orchids and shattered fir bark (2∶1; v/v) in a 5-cm diameter polyethylene planting bag; 5) each plantlet was potted in substrate mixture 2 containing commercial sand for orchids, sieved peat and shattered fir bark (2∶1∶1; v/v) in a 5-cm diameter polyethylene planting bag. Transplanted plantlets were grown in a greenhouse under a photosynthetic photon flux density (PPFD) of under 800 µmol m^−2^ s^−1^ natural light with sunshade nets. Plantlets were watered at 1- or 2-day intervals. After one month of acclimatization, plantlets were fertilized weekly with 150 mg l^−1^ 20-20-20 fertilizer (N-P-K; Peters Professional 20-20-20; The Scotts Co., Marysville, OH, USA). Average temperatures ranged from 20 to 32°C and humidity levels ranged from 70 to 98%. The percentage of plantlet survival was recorded 60 days after transplanting. Each experiment consisted of three independent replicates with 100 plantlets per replicate.

### Field establishment and ecorehabilitation

Field establishment and ecorehabilitation was conducted at three locations: 1) Yuanjiang Nature Reserve (23°40′N, 98°20′, 102°00′E, a natural habitat of *R. imschootiana* at an elevation of 480 m) in Yuanjiang county, Yunnan province; 2) Ehuangzhang Nature Reserve (21°55′N, 111°30′E, at an elevation of 480 m) in Yangchun, Guangdong province, the same elevation as the natural habitat; 3) Huolu Mountain Forest Park (23°18′N, 113°38′E, at an elevation of 85 m) in Guangzhou, Guangdong province, at a similar latitude as its natural habitat. *In vitro*-derived plantlets were potted on substrate mixture 2 containing commercial sand for orchids, sieved peat and shattered fir bark (2∶1∶1; v/v) in a greenhouse in South China Botanical Garden, Guangzhou, or were transplanted onto the trunks of *Pinus massoniana* trees by fully packaging roots with Chilean sphagnum moss and placing plantlets at the three locations in April, 2009. All plantlets were watered with a mist spray 1, 3, 7, 14, 21 and 28 days after ecorehabilitation, but were not watered thereafter. The survival, growth and development of all transplanted plants were monitored starting three months from August 20, 2009. The percentage of plantlet survival was recorded 360 days and 720 days after re-establishment in the wild. Each experiment consisted of three independent replicates with 100 plantlets per replicate.

### Culture conditions

Whenever special illumination requirements did not exist, then all cultures were incubated in 500-ml conical flasks closed with perforated rubber stoppers and plugged with cotton. Each flask contained 90 ml of medium solidified with 5.5 g l^−1^ agar (Huankai Microbial Sci. & Tech, Co., Ltd., Guangdong, China). Medium pH was adjusted to 5.8 with 1 mol l^−1^ KOH and 1 mol l^−1^ HCl before autoclaving at 121°C for 20 min at 1.06 kg cm^−2^. The CW used in these experiments was obtained from 6- to 7-month-old green coconuts from Hannan province, China and was filtered through one sheet of filter paper. Cultures were incubated at 25±1°C with a 16-h photoperiod under cool white fluorescent lamps delivering a PPFD of *ca.* 45 µmol m^−2^ s^−1^ [14].

### Data analysis

All experiments were conducted in a completely randomized design. Data was analyzed with Statistical Product and Service Solutions (SPSS) version 17.0 for Windows (Microsoft Corp., Washington, USA) using one-way analysis of variance (ANOVA) followed by Duncan's multiple range test (DMRT) at *P* = 0.05. Percentage data was arcsin transformed before subjecting it to ANOVA.

## Results

### Effect of basal media on germination *in vitro*

After 75 days of culture, seeds germinated on all 10 tested basal media, but the germination percentage differed (Table 2). Highest seed germination percentage (78.67%) occurred on 1/4 MS media, which was significantly higher than on 1/2 MS, MS, VW and Thomale GD media, but was not significantly higher than on 1/8 MS, KC, VW, Hyponex N016 and Hyponex N026 media. However, on 1/4 medium, 29.33% of protocorms developed to Stage 5, which was significantly higher than on all other media except for Hyponex N016 medium (25.33%). Only 54.27% of seeds germinated and 7.33% developed to Stage 5 on MS medium, which was significantly lower than on all other media. Therefore, 1/4 MS or Hyponex N016 media were the most appropriate basal media for seed germination and subsequent development of *R. imschootiana* protocorms among all 10 basal media tested.

**Table 2:** Effect of basal medium supplemented with 0.5 mg l^−1^ NAA, 10 g l^−1^ sucrose and 1.0 g l^−1^ AC on germination and development of *Renanthera imschootiana* 150 DAP seed after 75 days in culture.

Basal medium	Seedling development stage (%)					
	Stage 1	Stage 2	Stage3	Stage 4	Stage 5	Total germination (Stages 1–5)
1/8 MS	6.33±1.33 bc	12.33±1.45 a	11.67±0.88 c	19.00±2.08 b	24.33±1.76 b	73.67±1.78 ab
1/4 MS	6.00±1.00 c	12.00±1.00 a	13.67±0.88 bc	17.67±1.45 b	29.33±2.33 a	78.67±3.48 a
1/2 MS	6.00±1.15 c	11.00±1.00 ab	16.67±0.33 ab	12.10±1.46 a	19.33±1.20 cd	65.10±2.38 b*
MS	11.33±2.03 a	12.00±1.15 a	11.33±0.88 c	10.60±1.70 a	7.33±1.20 e	52.60±2.18 c*
KC	7.33±0.33 bc	12.33±1.45 a	17.00±1.15 ab	19.67±1.45 b	17.33±1.20 cd	73.67±1.26 ab*
VW	9.67±0.33 ab	11.33±0.88 ab	15.67±0.67 abc	21.00±1.73 b	14.67±1.45 d	72.33±1.69 ab*
RE	4.67±0.33 c	11.00±1.00 ab	18.33±2.03 a	18.00±2.08 b	16.67±0.88 d	68.67±2.40 b
Thomale GD	6.33±1.45 bc	9.67±0.88 ab	15.33±2.60 abc	19.67±2.19 b	18.00±1.53 cd	69.00±20.8 b
Hyponex N016	4.67±0.33 c	10.33±0.88 ab	13.67±1.86 bc	18.67±0.67 b	25.33±1.45 ab	72.67±1.45 ab
Hyponex N026	4.67±0.67 c	8.33±0.88 b	17.33±0.33 ab	17.33±0.33 b	21.67±0.88 bc	71.00±3.06 ab

For each treatment, approximately 300 seeds were cultured in a 500-ml culture flask containing 90 ml of medium. All experiments consisted of three independent replicates with 10 culture flasks per replicate. Values followed by different letters within a column are significantly different at *P*<0.05 (DMRT). Percentage data were arcsin transformed before being subjected to ANOVA. Each mean is based on microscopic observations. DAP, days after pollination; KC, Knudson's C medium; MS, Murashige and Skoog medium; NAA, α-naphthaleneacetic acid; RE, Robert Ernst medium; VW, Vacin and Went medium.

*Lin et al. (2008).

### Effect of degree of seed maturity on germination *in vitro*

**Figure 2:**
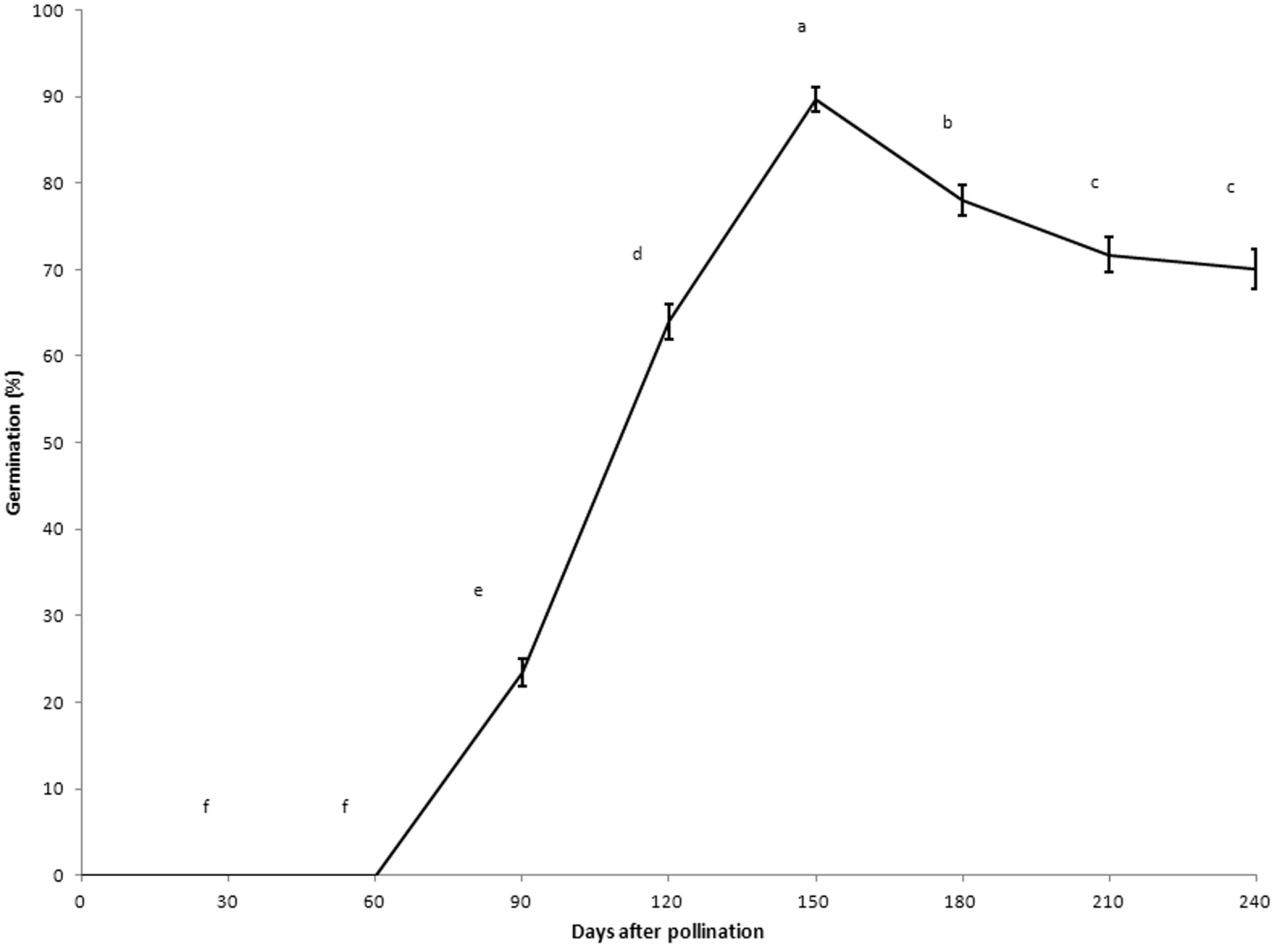
Effect of the degree of seed maturity on *in vitro* germination of *Renanthera imschootiana* on quarter-strength MS medium supplemented with 0.5 mg l^−1^ NAA, 20% CW, 1.0 g l^−1^ peptone, 10 g l^−1^ sucrose and 1.0 g l^−1^ AC 75 days after culture. Different letters indicate significant differences between days at *P*<0.05 (DMRT).

Seed germination percentage was significantly affected by the degree of seed maturity (Fig. 2). Immature (30 or 60 DAP) embryos or seeds, which were white, did not germinate because embryos had not developed completely. The highest seed germination percentage (89.67%) was observed in 150 DAP seeds on 1/8 MS medium at 75 days after culture, significantly higher than all other seeds of different ages. The seeds at 75 days after culture were initially grey-green in color but gradually became grey and brown by 210 and 240 DAP, respectively. Most capsules dehisced by 270 DAP. A mean of 5250 seeds could be recovered from each mature capsule, and the individual mean seed dry weight was 1.71 µg.

### Effect of sucrose concentration on seed germination

**Figure 3:**
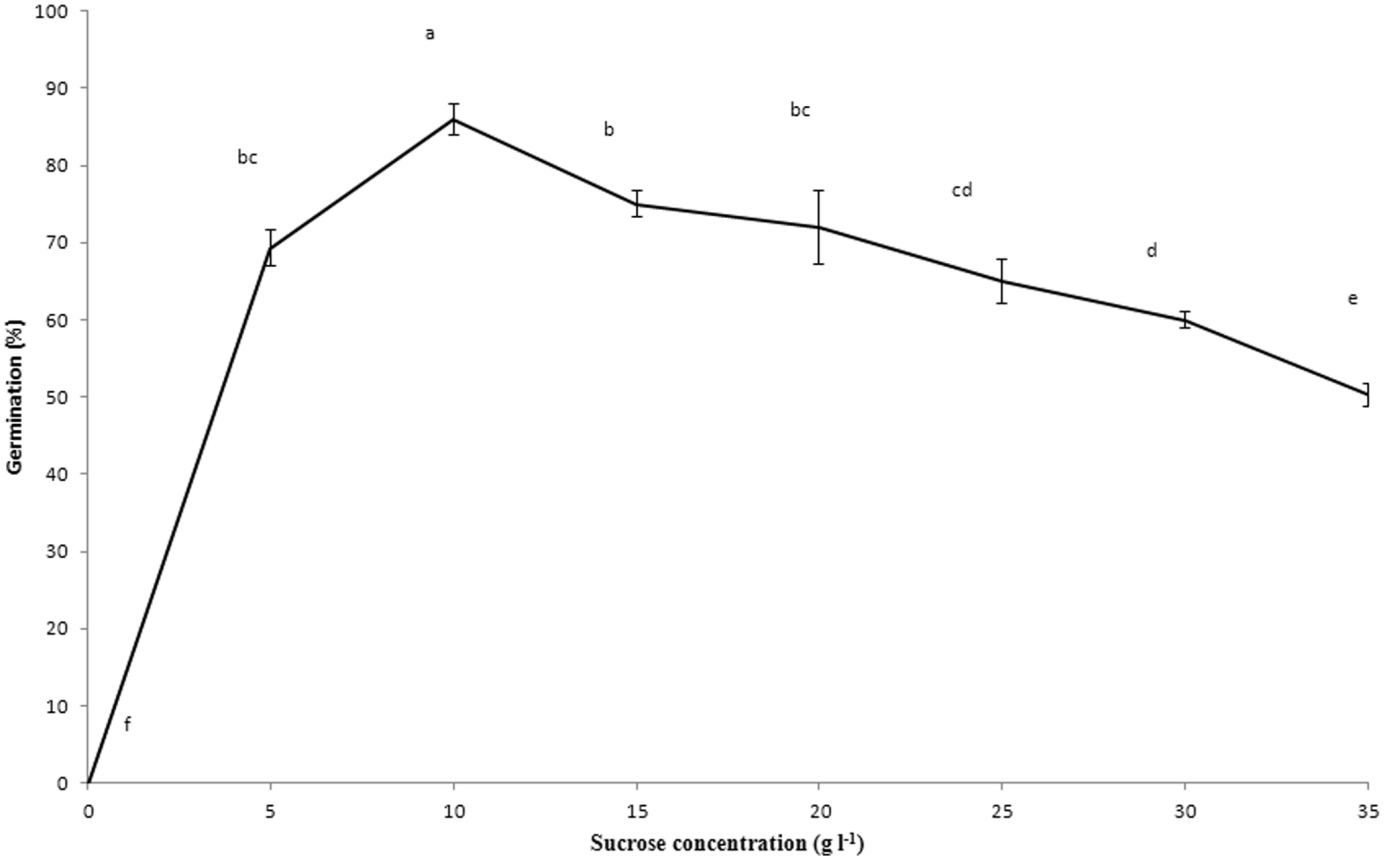
Effect of sucrose concentration on *in vitro* germination of *Renanthera imschootiana* on quarter-strength MS medium supplemented with 0.5 mg l^−1^ NAA, 20% CW and 1.0 g l^−1^ AC 75 days after culture. Different letters indicate significant differences between days at *P*<0.05 (DMRT).

Seed germination percentage was significantly affected by sucrose concentration (Fig. 3). The highest percentage of seed germination (86%) was observed in 150 DAP seeds on 1/4 MS medium supplemented with 10 g l^−1^ sucrose at 75 days after culture.

### Effect of organic amendments on seed germination

Seed germination percentage and protocorm development were affected by the kind and concentration of organic amendment used (Table 3). The percentage of seed germination was significantly lower on 1/4 MS without any organic amendments than on 1/4 MS containing 100 or 150 mg l^−1^ PH or any concentration of BH (50, 100, or 150 mg l^−1^). In contrast, when 1/4 MS contained any concentration of CW (10%, 15%, 20% or 25%), seed germination percentage was not significantly different to 1/4 MS without any organic amendments. However, seed germination percentage was significantly higher on 1/4 MS containing 25% CW in combination with 0.5 g l^−1^ peptone, than with other organic amendments or without any organic amendments. On the other hand, 41.10% of protocorms developed into Stage 5 seedlings on 1/2 MS containing 25% CW in combination with 0.5 g l^−1^ peptone which was significantly higher than all other treatments. When 1/4 MS contained 100 or 150 mg l^−1^ PH or any concentration of BH (50, 100, or 150 mg l^−1^), the percentage of protocorms that developed into Stage 5 was significantly lower on 1/4 MS without any organic amendments, while 1/4 MS without any organic amendments but containing 15% or 20% CW showed a significantly higher percentage of protocorms that developed to Stage 5.

**Table 3:** Effect of organic amendments on seed germination of *Renanthera imschootiana* at 150 DAP on 1/4 MS containing 0.5 mg l^−1^ NAA, 10 g l^−1^ sucrose and 1.0 g l^−1^ AC for 75 days in culture.

Organic amendments	Seedling development stage (%)					
	Stage 1	Stage 2	Stage 3	Stage 4	Stage 5	Total germination (Stages 1–5)
Control	6.00±1.00 gh	12.00±1.00 ab	13.67±0.88 def	17.67±1.45 cde	29.33±2.33 def	78.67±3.48 cd
10% CW	5.33±0.67 gh	8.67±0.67 bcde	14.00±1.53 def	21.23±2.30 abc	31.43±1.67 bcdef	80.67±3.31 bcd
15% CW	4.33±0.67 h	6.33±0.88 ef	16.23±3.67 bcde	19.57±1.27 bcd	35.10±1.65 bc	81.53±1.58 bcd
20% CW	3.67±0.33 h	3.67±0.67 f	20.33±1.20 abc	20.57±1.67 bcd	36.33±0.88 b	84.57±1.37 bc
25% CW	7.00±0.58 efgh	6.67±0.67 def	18.20±1.63 abcd	17.67±1.45 cde	30.00±1.15 cdef	79.53±1.47 cd
PH 50 g l^−1^	9.67±0.88 def	7.33±0.33 de	14.13±2.50 def	15.00±1.15 ef	28.77±1.47 ef	74.9±1.69 de
PH 100 g l^−1^	12.33±1.45 cd	8.33±0.33 bcde	6.67±1.37 gh	16.67±0.88 def	27.67±1.45 f	71.67±0.71 ef
PH 150 g l^−1^	14.00±1.15 c	12.00±1.15 ab	6.77±1.18 gh	16.67±1.20 def	17.90±1.64 g	67.33±2.91 fg
BH 50 g l^−1^	14.57±0.81 c	14.33±1.20 a	6.20±0.92 gh	16.67±0.88 def	20.23±1.36 g	72.00±2.08 ef
BH 100 g l^−1^	18.33±0.60 b	11.33±1.33 abc	6.83±0.83 gh	13.00±1.53 g	17.67±1.45 g	67.67±1.45 fg
BH 150 g l^−1^	26.23±3.40 a	12.00±3.05 ab	5.43±0.30 h	8.33±0.88 g	12.33±1.45 h	64.33±2.33 g
Tryptone 0.5 g l^−1^	11.67±0.88 cd	12.00±0.58 ab	12.43±1.37 ef	9.23±1.13 g	32.67±1.45 bcdef	78.00±2.65 cde
Tryptone 1.0 g l^−1^	8.00±0.58 efg	8.77±0.62 bcde	10.37±0.52 fgh	17.00±1.73 cdef	33.77±0.91 bcde	77.90±1.05 cde
Tryptone 1.5 g l^−1^	10.00±1.15 de	11.23±0.91 abc	7.00±0.58 gh	18.67±1.76 cde	30.67±1.45 cdef	77.57±1.37 de
Peptone 0.5 g l^−1^	5.00±0.58 gh	8.00±0.58 cde	11.67±1.52 efg	23.00±0.00 ab	33.00±1.73 bcdef	80.53±1.47 bcd
Peptone 1.0 g l^−1^	4.00±0.58 gh	6.67±0.67 def	11.60±1.22 efg	24.57±0.30 a	34.67±0.88 bcd	81.50±0.76 bcd
Peptone 1.5 g l^−1^	5.33±0.33 gh	9.00±0.58 bcde	15.00±1.53 def	20.00±0.58 bcd	30.33±1.45 cdef	79.67±0.88 cd
20% CW + peptone 0.5 g l^−1^	6.33±0.33 fgh	10.00±0.58 bcd	22.43±1.74 a	17.00±1.15 cdef	31.33±2.34 bcdef	86.90±2.48 b
20% CW+ peptone 1.0 g l^−1^	5.33±0.33 gh	11.00±1.53 abc	15.33±2.18 cdef	20.33±0.88 bcd	41.10±1.46 a	93.10±1.56 a
20% CW+ peptone 1.5 g l^−1^	8.00±1.15 efg	11.67±0.88 ab	20.83±1.17 ab	14.67±0.88 ef	29.33±2.33 def	84.43±2.35 bc

For each treatment, approximately 300 seeds (in three replicates) were cultured in a 500-ml culture flask containing 90 ml of medium. All experiments consisted of three independent replicates with 10 culture flasks per replicate. Values followed by different letters within a column are significantly different at *P*<0.05 (DMRT). Percentage data were arcsin transformed before being subjected to ANOVA. Each mean is based on microscopic observations. BH, banana homogenate; CW, coconut water; MS, Murashige and Skoog medium; NAA, α-naphthaleneacetic acid; PH, potato homogenate.

### Effect of BA concentration on PLB proliferation

PLB proliferation was significantly affected by the BA concentration (Table 4). However, from the 4th to 12th sub-cultures, the PLB proliferation ratio ranged between 2.70 and 2.95, which were not significantly different (data not shown). The highest PLB proliferation percentage (75.67%) and PLB proliferation ratio (2.88), the lowest PLB differentiation percentage (15.33%) and PLB necrosis percentage (9.00%) were observed on 1/4 MS medium supplemented with 1.5 mg l^−1^ BA, 0.5 mg l^−1^ NAA, 1.0 g l^−1^ peptone, 10 g l^−1^ sucrose, and 20% CW after 60 days of culture (Fig. 1G) in the 8th sub-culture. The PLB proliferation percentage and proliferation ratio were significantly higher than other treatments except for the PLB proliferation ratio on 1/4 MS medium supplemented with 2.0 mg l^−1^ BA and the same medium constituents.

**Table 4:** Effect of BA concentration on proliferation of *Renanthera imschootiana* PLBs on 1/4 MS medium with 0.5 mg l^−1^ NAA, 1.0 g l^−1^ peptone, 10 g l^−1^ sucrose, and 20% CW after 60 days in culture on the 8th sub-culture.

BA (mg l^−1^)	PLB proliferation (%)	PLB proliferation ratio	PLB differentiation (%)	PLB necrosis (%)
0	0 e	0 d	59.33±2.33 a	40.67±2.33 a
0.2	29.67±1.45 d	1.92±0.04 c	49.00±1.76 b	21.33±0.88 d
0.5	44.67±2.03 c	2.25±0.10 b	39.33±1.20 c	16.00±1.00 e
1.0	61.67±4.41 b	2.55±0.10 b	27.33±1.45 d	11.00±3.05 ef
1.5	75.67±2.33 a	2.88±0.16 a	15.33±1.45 e	9.00±1.00 f
2.0	62.67±1.86 b	3.11±0.07 a	9.67±1.45 f	27.67±0.88 c
2.5	60.33±2.19 b	2.32±0.10 b	5.33±0.88 f	34.33±1.33 b

Means ± SE of 300 replicates; different letters within each column are significantly different at *P*<0.05 (DMRT).

### Effect of NAA and banana homogenate concentration on PLB differentiation

PLB differentiation was significantly affected by NAA and BH concentration (Table 5). The highest percentage of plantlet formation (95.67%) was observed on 1/4 MS medium supplemented with 1.0 mg l^−1^ NAA, 100 g l^−1^ BH and 1.0 g l^−1^ peptone, 10 g l^−1^ sucrose, 10% CW and 1.0 g l^−1^ AC after 60 days of culture (Fig. 1H), which was significantly higher than all other treatments. The percentage of plantlet formation on 1/4 MS medium containing all tested combinations of NAA and BH, or containing 50 g l^−1^ BH was significantly higher than the control (1/4 MS medium without NAA and BH), while the percentage of plantlet formation on 1/4 MS medium containing 1.0, 1.5, or 2.0 mg l^−1^ NAA was significantly lower than the control.

**Table 5:** Effect of NAA and banana homogenate (BH) concentration on differentiation of *Renanthera imschootiana* PLBs on 1/4 MS medium containing with 1.0 g l^−1^ peptone, 10 g l^−1^ sucrose, 10% CW and 1.0 g l^−1^ AC after 60 days in culture.

NAA (mg l^−1^)	BH (g l^−1^)	PLB necrosis (%)	PLB proliferation (%)	Plantlet formation (%)
0	0	19.67±0.88 b	5.33±0.88 fg	75.00±1.73 d
0.5	0	7.33±1.20 efg	20.67±2.33 c	72.00±1.15 d
1.0	0	2.66±0.33 h	30.00±1.15 b	67.33±1.45 e
1.5	0	3.33±0.33 h	39.00±2.08 a	57.67±1.76 f
2.0	0	14.33±1.20 c	30.67±2.33 b	55.00±1.15 f
0	50	8.33±0.88 ef	8.00±1.15 f	83.67±1.67 c
0	100	12.67±1.45 cd	13.33±0.88 d	74.00±2.00 d
0	150	15.00±1.73 c	8.67±0.67 df	76.3±31.86 d
0	200	24.67±1.45 a	21.67±2.18 c	53.67±0.88 f
0.5	50	5.00±0.58 fgh	13.00±1.73 de	82.00±1.15 c
0.5	100	3.67±0.67 h	13.67±1.76 d	82.67±1.76 c
0.5	150	7.33±1.33 efg	9.67±1.20 def	83.00±1.53 c
1.0	50	4.00±0.58 gh	5.00±0.58 fg	91.00±1.00 b
1.0	100	2.00±0.58 h	2.33±0.33 g	95.67±0.67 a
1.0	150	10.33±1.45 de	5.33±0.33 fg	84.33±1.76 c

All experiments consisted of three independent replicates with 10 culture flasks per replicate and 20 PLBs per flask. Values followed by different letters within a column are significantly different at *P*<0.05 (DMRT).

### Effect of organic amendments on plantlet growth *in vitro*

Plantlet growth *in vitro* was significantly affected by NAA and BH concentrations (Table 6). Following the assessment of all parameters (shoot height, number of roots, length of longest root, fresh weight/plantlet, growth status), Hyponex N016 medium supplemented with 0.5 mg l^−1^ NAA, 150 g l^−1^ BH, 1.0 g l^−1^ peptone, 20 g l^−1^ sucrose, 10% CW and 1.0 g l^−1^ AC was found to be most suitable for plantlet growth *in vitro* since it resulted in the tallest shoots, most roots, longest roots and highest fresh weight (Fig. 1I). When Hyponex N016 medium was supplemented with a higher concentration of BH (200 g l^−1^) or without NAA and BH, the growth status of plantlets was poor.

**Table 6:** Effect of NAA and banana homogenate (BH) concentration on *Renanthera imschootiana* plantlet growth on Hyponex N016 medium with 1.0 g l^−1^ peptone, 20 g l^−1^ sucrose, 10% CW and 1.0 g l^−1^ AC after 60 days in culture.

NAA (mg l^−1^)	BH (g l^−1^)	Shoot height (cm)	No. of roots	Length of longest root (cm)	Fresh weight/plantlet (g)	Growth status*
0	0	2.64±0.06 gh	2.61±0.06 h	2.69±0.09 f	0.32±0.12 g	+
0.5	0	3.01±0.08 cde	2.81±0.10 gh	2.97±0.08 def	0.41±0.02 fg	++
1.0	0	3.14±0.15 bcd	2.99±0.08 efg	3.17±0.06 cde	0.61±0.06 de	+++
1.5	0	2.87±0.21 def	3.17±0.06 bcdef	3.80±0.14 ab	0.52±0.04 ef	++
2.0	0	2.91±0.12 def	3.03±0.12 efg	3.29±0.13 cd	0.46±0.03 f	++
0	50	3.02±0.09 cde	2.82±0.12 fgh	2.78±0.12 f	0.46±0.03 f	+
0	100	2.98±0.12 cdef	3.05±0.13 defg	2.98±0.12 def	0.52±0.05 ef	++
0	150	2.69±0.09 fgh	3.38±0.09 bcd	3.30±0.09 cd	0.65±0.03 cd	++
0	200	2.51±0.09 h	2.92±0.11 fgh	2.96±0.10 def	0.46±0.04 f	+
0.5	100	3.42±0.06 ab	3.42±0.14 bc	3.50±0.08 bc	0.71±0.04 bcd	+++
0.5	150	3.63±0.11 a	3.83±0.12 a	4.13±0.15 a	0.93±0.04 a	+++
0.5	200	3.21±0.07 bcd	3.43±0.12 bc	3.82±0.16 ab	0.79±0.04 b	+
1.0	100	3.28±0.10 bc	3.46±0.13 b	4.03±0.15 a	0.75±0.03 bc	+++
1.0	150	3.42±0.09 ab	3.30±0.09 bcde	3.92±0.14 a	0.79±0.02 b	+++
1.0	200	2.78±0.09 fgh	3.11±0.06 cdefg	2.80±0.14 df	0.71±0.04 bcd	+

* +, ++, +++ represents poor, normal, and good growth, respectively (see text for detailed explanation). All experiments consisted of three independent replicates with 10 culture flasks per replicate and 20 PLBs per flask. Values followed by different letters within a column are significantly different at *P*<0.05 (DMRT).

### Greenhouse acclimatization

Plantlets grew vigorously 30 days after transplanting. The highest percentage of plantlet survival (Fig. 1J; 95%) observed on Chilean sphagnum moss 60 days after transplanting, was significantly higher than plantlets fixed on fir bark blocks or in the two substrate mixtures (Table 7). However, the roots of transplanted seedlings hardly elongated or formed any new roots on Chilean sphagnum moss. Moreover, fir bark blocks with Chilean sphagnum moss was also suitable for transplanting seedlings, and growth of plantlets was not significantly different than on Chilean sphagnum moss in which the roots elongated rapidly, with roots elongating from 3–4 to 5–6 cm. About 10,000 plantlets germinated from seed and were successfully planted within two years. In the same period, almost all seedlings were successfully acclimatized to greenhouse conditions. These plantlets can be used for ornamental, ecorehabilitation and conservation purposes.

**Table 7:** Survival of *Renanthera imschootiana* plantlets grown on different substrates after transplanting for 60 days.

Media of transplanting	Survival (%)
Fixed on fir bark blocks	75.00±3.00 d
Chilean sphagnum moss	95.00±1.73 a
Fixed on fir bark blocks packaged roots with Chilean sphagnum moss	89.33±1.76 ab
Substrate mixture 1*	81.33±2.33 cd
Substrate mixture 2**	87.67±1.45 bc

Means ± SE of 300 replicates with the different letters within columns are significantly different at *P*<0.05 (DMRT).

* Substrate mixture 1: commercial sand for orchids+shattered fir bark (2∶1; v/v).

** Substrate mixture 2: commercial sand for orchids+sieved peat+shattered fir bark (2∶1∶1; v/v).

### Field establishment and ecorehabilitation

After *in vitro* plantlets were potted on substrate mixture 2 in a greenhouse in South China Botanical Garden, survival was 100% after three years. The *in vitro* plantlets were transplanted onto tree trunks and one year after transplantation, highest survival was 84.67% at Yuanjiang Nature Reserve from seedlings transplanted for 360 days which was significantly higher than at the two other reintroduction locations but not significantly higher than seedlings transplanted for 180 days at the same location (Table 8). Two years after transplantation, the highest survival rate was 79.67% at Yuanjiang Nature Reserve from seedlings transplanted for 360 days, which was significantly higher than at Ehuangzhang Nature Reserve, but not significantly higher than seedlings transplanted for 180 days at the same location or seedlings transplanted for 360 days at Huolu Mountain Forest Park. Three years after transplantation, 20% of plants that survived flowered from seedlings transplanted for 360 days at all three reintroduction locations. However, no plants produced fruits or seeds.

**Table 8:** Survival rates of different ages of transplanted *Renanthera imschootiana* seedlings after 360- or 720-day reintroduction.

Reintroduction location	Age of transplanted seedlings (days)	Survival after 360 days of reintroduction (%)	Survival after 720 days of reintroduction (%)
Huolu Mountain Forest Park, Guangzhou	180	71.67±3.76 b	60.00±2.89 cd
	360	75.00±2.87 b	71.33±20.8 ab
Yuanjiang Nature Reserve, Yuanjiang	180	79.67±2.03 ab	76.67±4.06 a
	360	84.67±2.03 a	79.67±2.40 a
Ehuangzhang Nature Reserve, Yangchun	180	62.67±1.45 c	57.67±1.45 d
	360	73.33±2.40 b	66.67±2.40 bc

Means ± SE of 300 replicates with the different letters are significantly different at *P*<0.05 (DMRT).

## Discussion

### Effect of basal media on seed germination and seedling development *in vitro*

In this study, *R. imschootiana* seed germination and seedling development were considerably affected by the choice of medium and medium additives. Many orchid species prefer medium with a low salt and nitrogen for seed germination and PLB formation [14], [21]–[23]. *R. imschootiana* showed significantly higher seed germination on 1/4 MS than on MS or 1/2 MS medium possibly because of the high salt concentration of the latter two media although seed germination on 1/4 MS was not significantly higher than that on 1/8 MS medium. Zeng et al. [14] reported that seed germination of *Paphiopedilum wardii* on 1/2 MS was significantly higher than on 1/4 MS or MS medium. This indicates that the effect of basal media on seed germination is most likely to be genotype-dependent in orchids. Wu et al. [5] reported the use of VW medium for the efficient regeneration of *Renanthera* Tom Thumb ‘Qilin’ from leaf explants. In contrast, in the present study, 1/4 MS medium was more suitable for seed germination, seedling development, and regeneration of plants through PLBs than VW medium (data not shown).

### Effect of seed maturity degree on germination *in vitro*

Asymbiotic seed germination of fully mature terrestrial orchid seeds is often difficult and immature seeds need to be germinated more readily [14], [24]–[26], while fully mature epiphytic orchid seeds have a high seed percentage[10],. *R. imschootiana* is an epiphytic orchid, although the percentage seed germination of fully mature seeds (240 DAP) was also high (70%). However, the germination of fully mature seeds was significantly lower than the two earlier development stages, namely 150 and 180 DAP. Factors that affect the low germination percentage of mature terrestrial seeds include an impermeable testa [21], the presence of chemical inhibitors such as abscisic acid (ABA), or the lack of certain hormones that promote germination [26]. In addition, Lee et al. [25] found that the suspensor may be the major site for nutrient uptake by the developing embryo of *Paphiopedilum delenatii* and that, during embryo development, the suspensor disappears, and water and nutrient uptake mainly depend on the permeability of the testa and result in a decrease in seed germination percentage. Mweetwa et al. [27] noted that treatments with calcium hypochlorite could promote seed germination in *Phalaenopsis* because the testa was eroded and became more permeable to water and nutrients. However, as for terrestrial orchids, mature seeds of epiphytic orchids may have a greater potential for propagation and storage [28].

### Effect of organic amendments on germination and plantlet growth in vitro

Organic additives such as tryptone, peptone, CW, BH, PH, and others are commonly added to orchid media to promote seed germination, seedling growth and rooting [5]–[6], [9], [14], [20], [29]–[35], which generally consist of low molecular weight proteins, amino acids, vitamins and plant growth substances, which are able to enhance plant growth by providing plant cells with a readily available source of nitrogen [36]. In the present study, tryptone and peptone (0.5, 12.0, or 1.5 g l^−1^), or CW (10%, 15%, 20%, or 25%) in media did not significantly affect seed germination. In contrast, Pierik et al. [37] reported that tryptone (1.5, 2.0, or 2.5 g l^−1^) significantly promoted seed germination and the further development of *Paphiopedilum ciliolare* seedlings. Zeng et al. [14] reported that seed germination percentage of *Paphiopedium wardii* increased significantly when immature seeds were cultured on 1/2 MS medium supplemented with 0.5, 1.0, or 1.5 g l^−1^ peptone. In the present study, CW, when combined with peptone, was optimum for seed germination. 1/4 MS containing 20% CW and 1.0 g l^−1^ peptone was most suitable for germination than other concentrations of CW in combination with peptone, or when 1/4 MS medium contained only tryptone, peptone, CW, BH, or PH. The enhancing effect of CW may be because it contains many different types of biochemicals, including amino acids, vitamins, sugar, minerals and phytohormones [38]–[39] as well as various inorganic ions such as phosphorus, magnesium, potassium, and sodium [40]. CW is commonly added to orchid media to stimulate seed germination or PLB formation [10], [14], [30], [41]–[43].

When the media contained BH, or a high concentration of PH (100 or 150 g l^−1^), seed germination percentage was significantly lower than the control (without organic additives). However, a suitable concentration of BH was beneficial for the differentiation of PLBs and plantlet growth at 100 or 150 g l^−1^, respectively. Arditti and Ernst [44] reported that BH is a rich source of natural cytokinins which inhibit culture initiation but promotes the differentiation and growth of shoots at later stages. The same could be observed in the present study in which the inhibitory effect on germination caused by a high concentration of BH may be because such medium contains too much sugar or other substances that would allow for the effective germination of seed, even though the same medium is suitable for plantlet growth *in vitro* [14], [37].

Among the plant growth regulators, BA (equivalent to 6-benzylaminopurine, or BAP) [45] plays an important role in plant regeneration in the tissue culture of orchids [32]. In our experiment, BA was used to induce PLB proliferation, and 1/4 MS medium with 1.0 mg l^−1^ BA, 0.5 mg l^−1^ NAA, 1.0 g l^−1^ peptone, 10 g l^−1^ sucrose, and 20% CW was the most effective medium. When the medium did not contain BA, protocorms or PLBs could not proliferate (Table 4).

There are many reports in which the addition of AC to medium improved orchid seed germination and plantlet growth *in vitro* [46]–[48]. AC may improve aeration, add microelements, affect substrate temperature, establish polarity, or absorb toxic substances, including phenolics, when employed in media [46], [48]–[49].

### Field establishment and ecorehabilitation

A similar environment to that found in nature is likely to be suitable for the growth of orchid plantlets, even in greenhouse conditions [20]. *R. imschootiana* grows only as an epiphyte on the trunks of trees in natural habitats. In the present study, *in vitro* plantlets were fixed to fir bark blocks or placed in substrate mixtures in pots to assess the differences in survival as a result of the planting method employed. The survival of *in vitro* plantlets whose roots were packaged in Chilean sphagnum moss and fixed onto fir bark blocks was not significantly different to that of plantlets potted in Chilean sphagnum moss or in substrate mixture 2, which contains commercial sand for orchids, sieved peat and shattered fir bark. However, the result was different for *Nothodoritis zhejiangensis* [20]. In that study, the survival of plantlets planted on fir bark blocks was significantly higher than in pots with Chilean sphagnum moss or a sand/peat/fir bark mixture 180 days after transplanting.

The ideal place to conserve plant germplasm and to promote biodiversity is in the wild, *in situ*, where a large number of species present in viable populations can persist in their natural habitats with their natural associated ecological interactions [50]. Although the survival of the 6-month *in vitro* plants was 100% when they were potted on substrate mixture 2 after two years, reintroduction of native species, especially of species that are rare and threatened, has become increasingly important in conservation worldwide for recovery of rare species and restoration purposes, which also has become an important tool for biodiversity conservation [51]–[53]. Some studies have reported the outcome of reintroduction efforts in plant species [50]. Seeni and Latha [32] reported the ecorehabilitation of the endangered Blue Vanda (*Vanda coerulea*) in which survival exceeded 70%. Zeng et al. [14] and Ren et al. [53] reported successful reintroduction of *Paphiopedilum wardii* and *Tigridiopalma magnifica* in their natural habitats and alien forest habitat, respectively. In the percent study, *R. imschootiana* could not be found at a single habitat in Yuanjiang, Yunnan, in China. However, this study re-established *R. imschootiana* plants in the wild, including in habitats at Yuanjiang in Yunnan and in two alien forest habitats at Huolu Mountain Forest Park and Ehuangzhang Nature Reserve in Guangdong. The reintroduced seedlings from direct seed germination is conducive to biodiversity conservation because seed-derived progeny, unlike clonal plants derived from micropropagation - or the mass production from vegetative parts – are genetically heterogeneous, and would thus allow a higher percentage of the individuals within a population to survive and outcross [14]. One of the criteria for the successful reintroduction of a species is that the introduced individuals complete their full life cycle and give rise to a self-sustaining, regenerating population [53]. Although the survival of *R. imschootiana* plantlets was high after 36 months at three locations, including Huolu Mountain Forest Park and Ehuangzhang Nature Reserve, which lie outside the historical range of the species, and three years after ecorehabilitation, only 20% of plants that survived could flower at all three reintroduction locations. However, no plants produced fruits or seeds possibly due to the lack of suitable pollinators (insects) because *R. imschootiana* cannot be pollinated without insects in the greenhouse. Further research on successful reintroduction is needed, especially in new habitats, which can demonstrate that future human-assisted migration of this species, for example in the face of climate change, is possible. In addition, plantlets derived from PLB proliferation can be used for short- or long-term *in vitro* conservation of germplasm, but studies related to genetic stability and somaclonal variation should be conducted.

In conclusion, this study reports a holistic and practical procedure for asymbiotic germination, *in vitro* seedling culture and a regeneration system through PLBs, greenhouse acclimatization, as well as the re-establishment and ecorehabilitation of *R. imschootiana* plants in the wild. The procedure may also be useful for the conservation and commercial production of other threatened orchid species.

## Supporting Information

File S1 **Raw Data comprising effect sizes and sample sizes of all empirical quantitative articles.** Note: the complete data set is available on request from the corresponding author SZ.
(RAR)
